# Genetic variants of numb gene were associated with elevated total cholesterol level and low density lipoprotein cholesterol level in Chinese subjects, in Xinjiang, China

**DOI:** 10.1186/s13000-015-0373-2

**Published:** 2015-08-12

**Authors:** Mayila Abudoukelimu, Zhen-Yan Fu, Yang Xiang, Yi-Tong Ma, Qing Zhu, Minawaer Abudu, Dilare Adi, Yi-Ning Yang, Xiao-Mei Li, Xiang Xie, Fen Liu, Bang-Dang Chen

**Affiliations:** Department of Cardiology, the First Affiliated Hospital of Xinjiang Medical University, Urumqi, 830001 People’s Republic of China; Xinjiang Key Laboratory of Cardiovascular Disease Research, Urumqi, 830001 People’s Republic of China; The First Affiliated Hospital of Xinjiang Medical University, Urumqi, 830001 People’s Republic of China; Present address: Department of Cardiology, the First Affiliated Hospital of Xinjiang Medical University, Li Yu Shan South Road 137, Urumqi, 830001 People’s Republic of China; Present address: Xinjiang Key Laboratory of Cardiovascular Disease Research, Li Yu Shan South Road 137, Urumqi, 830001 People’s Republic of China

## Abstract

**Background:**

Hypercholesterolemia is one of the most common risk factors for Coronary Artery Disease (CAD), which is the leading cause of death worldwide. As Numb is an important regulating factor regarding intestinal cholesterol absorption and plasma cholesterol level, the aim of the present study is to investigate the relationship between human Numb gene polymorphism and cholesterol level in Chinese subjects.

**Methods:**

All participants came from the First Affiliated Hospital of Xinjiang Medical University (Male: 1052 and Female: 596), and four tagging SNPs (rs2108552, rs12435797, rs1019075 and rs17781919) of Numb gene were genotyped by using TaqMan^®^ assays and analyzed in an ABI 7900HT Fast Real-Time PCR System. Further, general liner model was applied for assessing the relationship between cholesterol level and genotypes.

**Results:**

By analyzing a dominant model, recessive model and an additive model, we have found that SNP rs2108552 was associated with total cholesterol (TC) and low density lipoprotein-cholesterol level (LDL-C) (*P* = 0.000 and *P* = 0.007; *P* =0.042 and *P* =0.009; *P* = 0.006 and *P* = 0.030). C allele of SNP rs17781919 had significantly lower plasma TC level (3.46 ± 0.74 mmol/L vs 4.27 ± 1.1 mmol/L) and LDL-C level (0.98 ± 0.55 mmol/L vs 2.64 ± 0.93 mmol/L) when compared with T allele. Additionally, SNP rs12435797 was associated with TC level and SNP rs1019075 was associated with LDL-C level by analyses of a dominant model, recessive model and an additive model (*P* = 0.000, *P* = 0.005 and *P* = 0.004; *P* = 0.016, *P* = 0.008 and *P* = 0.033). Further, the association of rs2108552, rs12435797, rs1019075 and rs17781919 with aforementioned different kinds of cholesterol levels remained statistically significant after multivariate adjustment of ethnicity, gender, age, smoking and obesity.

**Conclusions:**

Our results indicated that both rs2108552 and rs17781919 in the Numb gene were associated with total cholesterol level and density lipoprotein-cholesterol level in Chinese subjects.

## Background

Coronary artery disease (CAD) is the leading cause of morbidity and mortality in developed countries, and becoming increasingly prevalent in developing countries [1–3]. Regarding Coronary artery disease, strong evidence indicates that the risk increasement of CAD [4–7] is closely related to the rise of plasma cholesterol level.

Studies have demonstrated that a reduction in plasma total cholesterol (TC) and triglycerides (TG) concentration independently reduces the risk of CAD [8, 9]. In addition, convincing data also shows that 1 % decrease in low density lipoprotein-cholesterol (LDL-C) concentration reduces the risk of CAD for approximately 1 % [10], and 1 % increase in high density lipoprotein-cholesterol (HDL-C) concentration reduces the risk of CAD for approximately 2 % [11]. Further, other than being determined by environmental factors, the levels of plasma cholesterol are also influenced by the genetic constitution of each individual, such as single nucleotide polymorphisms (SNPs) [12–15].

Numb is a member of clathrin-associated sorting proteins, and combines with several other endocytic proteins [16–19]. Pei-Shan Li et al. [20] has revealed that Numb is an essential regulating factor for maintaining human cholesterol homeostasis. Numb regulates cholesterol absorption, and thus plays a crucial role in the development of atherosclerosis and indicated that pharmacologically targeting the Numb-NPC1L1 interaction could be a way to decrease cholesterol absorption and cholesterol level. Further, Jian Wei et al. [21] has confirmed that there does exist a remarkable correlation between Numb polymorphism G595D (rs17781919),-a nonsynonymous variant in the coding region of Numb gene- and low concentration of LDL-C among humans. They demonstrated that rs17781919 influences Numb activity of driving NPC1L1 internalization and decreases cholesterol absorption and leading to low plasma cholesterol level.

Unfortunately, the relationship between human Numb gene and lipid profile has not been thoroughly studied yet. Therefore, the aim of the present study is to reveal the relationship between some genetic variants of human Numb gene and lipid profile among Chinese population in Xinjiang, China, hoping to provide additional information to characterize the genetic factors that influence plasma cholesterol levels.

## Methods

### Subjects

Altogether, 1742 (Male: *n* = 1117; Female: *n* = 625) were randomly selected from the First Affiliated Hospital of Xinjiang Medical University that belong to a time period ranges from January 2007 to December 2013 for DNA analysis. The inclusion and exclusion criteria was such as: The analysis presented in this study was based on 1648 subjects (Male: *n* = 1052; Female: *n* = 596) who had passed the eligibility criteria and had complete data on Numb genotype. Further, all of these subjects live in Xinjiang Uighur Autonomous Region of China, and none of them suffers from impaired malignancy, connective tissue disease, renal function, valvular disease or chronic inflammatory disease. Moreover, subjects also are free from thyroid disease, or any history of taking lipid-lowering drugs. In addition we excluded 92 hypercholesterolemia (fasting plasma TC ≥ 6.26 mmol/L or fasting plasma LDL-C ≥ 4.14 mmol/L [22]) patients during the analysis.

### Lifestyle measurements and blood biochemical analysis

Height and body weight were measured as described previously [23], and body mass index (BMI) was calculated by dividing the weight in kilogram to the square of height in meter. The status of smoking was described as smokers (including current and ex-smokers) or non-smokers. Further, WHO Asia-Pacific Area criterion- BMI ≥25 kg/m^2^ was used to define obesity as described in detail previously [23]. Finally, blood urea nitrogen (BUN), creatinine (Cr), uric acid, glucose, total cholesterol (TC), triglyceride (TG), low density lipoprotein-cholesterol (LDL-C), and high density lipoprotein-cholesterol (HDL-C) were measured by using chemical analysis equipment (Dimension AR/AVL Clinical Chemistry System, Newark, NJ) in Clinical Laboratory Department of the First Affiliated Hospital of Xinjiang Medical University [24, 25].

### Ethical approval of the study protocol

All participants have given their written informed consent and explicit permission for DNA analysis as well as for the collection of relevant clinical data. This study was approved by the Ethics Committee of the First Affiliated Hospital of Xinjiang Medical University (Urumqi, China) and was conducted by strictly following the requirements of the Declaration of Helsinki.

### SNP selection

The human Numb gene includes phosphotyrosine-binding (PTB) domain and proline-rich region (PRR) domain [26–29] and has four transcript variants that encode four different isoforms, which are p72 (PTBLPRRL), p71 (PTBSPRRL), p65 (PTBSPRRS), and p66 (PTBLPRRS). Isoform p72 of the Numb gene, which has the longest length among the four isoforms, consists of 651 amino acids. It is located on chromosome 14q24.3 and contains 13 exons which are further separated by 12 introns. There are 3781 different kinds of SNPs of human Numb gene as listed in the National Center for Biotechnology Information SNP database (http://www.ncbi.nlm.nih.gov/SNP). For the current study, we have screened the HapMap phase I & II database and Haploview 4.0 software for the tag SNPs of Numb gene and selected three SNPs (rs2108552, rs12435797, and rs1019075). Meanwhile, we also included rs17781919 from the Numb gene which was associated with LDL-C [21]. SNPs with relatively high minor allele frequencies (MAFs) have been shown to be useful as genetic markers in genetic association studies [30] and all four tag SNPs that selected for the present study had a MAF of ≥0.1. In addition, linkage disequilibrium (LD) is the non-random association of alleles at different loci, and we described LD patterns with r^2^ ≥ 0.8 as a cut-off for the tag SNPs [31].

### Genotyping

Blood samples were taken from all participants by using anticoagulant ethylene diaminetetraacetic acid (EDTA) tube, and standard phenol-chloroform method was used to extract genomic DNA from peripheral leukocytes [32]. Further, genotyping was undertaken by using TaqMan^®^ assays from Applied Biosystems following the manufacturer’s suggestions and analyzed in an ABI 7900HT Fast Real-Time PCR System [33], and the detailed information of the TaqMan SNP Genotyping method was as follows. Allele-specific fluorogenic probes were hybridised to the template in the first step of the 5′ nuclease assay. Then, during the polymerase chain reaction (PCR), the 5′ nuclease activity of the Taq polymerase made it possible for discrimination. In addition, the probes include a 3′ minor groove binding group that hybridises to single-stranded targets and has greater sequence specificity when compared to the original DNA probes. This reduces nonspecific probe hybridization which leads to low background fluorescence for the 5′ nuclease PCR assay (TaqMan; Applied Biosystems). Cleavage results in the increased emission of a reporter dye. Two unlabeled PCR primers and two allele-specific probes were required for each 5′ nuclease assay. At the 5′ end, each probe is labeled with two reporter dyes. In the present study, VIC and FAM were used as the reporter dyes. The probes and primers used in TaqMan^®^SNP Genotyping Assays (ABI) were selected according to the information on ABI website (http://www.appliedbiosystems.com/absite/us/en/home.html). Finally, PCR amplification was performed by using 0.05 μL probes, 3 μL of TaqMan Universal Master Mix, 1 μL DNA, and 1.95 ddH2O in a 6 μL final reaction volume. In addition, thermal cycling conditions for PCR amplification were 95 °C for 10 min, 45 cycles of 95 °C for 10 s and 60 °C for 1 min. Moreover, thermal cycling was undertaken by using Applied Biosystems7900HT Standard Real-Time PCR System, and all 96 well plates were read according to Sequence Detection Systems (SDS) automation controller software v2.3 (ABI).

### Quality control

There was at least one negative and one positive control per 96-well DNA plate in our assays. We have found 100 % concordance between the genotyped duplicate samples (10 %) for each of the SNPs by testing the accuracy of the genotyping. The rate of genotyping success for each SNP was 98 %.

### Statistical analysis

All statistical analyses were performed via using SPSS16.0 software for Windows (SPSS Institute, Chicago, IL, USA). Further, Hardy-Weinberg equilibrium was assessed by *χ*2 analysis for all subjects, and all of the continuous variables were expressed via mean ± standard deviation. Furthermore, differences among the frequency of obesity, smoking and Numb genotypes were analyzed through *χ*2 test or Fisher’s exact test. Finally, after adjusting confounding variables, general linear model analysis was undertaken to test the association between Numb genotypes and lipid profile. In addition, *P* < 0.05was considered as a criterion for statistical significance.

## Results

### Characteristics of the subjects

The study cohort includes 1648 subjects (1052 male, 596 female), and all of them were selected from Out-patient and In-patient department of the First Affiliated Hospital of Xinjiang Medical University that belong to a time period ranges from January 2007 to December 2013.

### Results of outcome measures

1. The clinical and metabolic characteristics of the study population are shown separately for male and female in Table 1. In females, plasma concentration of BUN is higher than to males (*P* = 0.036). Further, in males, plasma concentration of creatinine, prevalence of smoking is higher than females (all *P* = 0.000). In addition, there were no significant differences between males and females on plasma concentration of uric acid, glucose, TC,TG, LDL-C and HDL-C or prevalence of obesity (All *P* > 0.05).

**Table 1:** Clinical and metabolic characteristics of subjects

Risk factor	No. (%) or mean (SD)			
	Total	Male	Female	*P* value
Age (years)	56.33 (9.83)	55.28 (10.09)	58.22 (9.03)	0.000^*^
BUN (mmol/L)	5.07 (1.82)	5.01 (1.85)	5.20 (1.78)	0.036^*^
Cr (mmol/L)	71.04 (17.18)	72.26 (17.29)	68.98 (16.79)	0.000^*^
Uric acid (mmol/L)	313.70 (89.96)	316.76 (87.43)	308.51 (93.79)	0.078
Glucose (mmol/L)	5.68 (2.43)	5.74 (2.41)	5.58 (2.39)	0.189
Total cholesterol (mmol/L)	4.27 (1.11)	4.33 (1.18)	4.23 (1.07)	0.083
Triglyceride (mmol/L)	1.97 (1.49)	2.01 (1.81)	1.94 (1.28)	0.401
LDL-C (mmol/L)	2.65 (0.91)	2.69 (0.99)	2.62 (0.86)	0.188
HDL-C (mmol/L)	1.05 (0.55)	1.04 (0.55)	1.06 (0.54)	0.378
Obesity (%)	1026 (63.1)	641 (61.6)	385 (66.0)	0.077
Smoking (%)	1031 (62.4)	737 (69.9)	293 (49.2)	0.000^*^

Continuous variables are expressed as mean ± SD. Categorical variables are expressed as percentages

The *P* value of the continuous variables was calculated by the independent samples *t* test. The *P* value of the categorical variables was calculated by *x* ^2^ test

*BUN* blood urea nitrogen, *Cr* Creatinine, *LDL-C* low density lipoprotein-cholesterol, *HDL-C* high density lipoprotein-cholesterol

^*^ *P* < 0.05
2. The distribution of genotypes of SNPs (all genotyped frequencies of SNPs were in Hardy-Weinberg equilibrium, data not shown) of Numb gene is shown in Table 2. We observed that there were no significant differences between males and females for 4 SNPs (All *P* > 0.05; genotypes, dominant, recessive and additive models). In addition, for the alleles, we found that there was significant difference between males and females for SNP rs12435797 (*P* = 0.031). Further, we found that there was no significant difference between males and females for rs2108552, rs1019075, and rs17781919 (All *P* > 0.05).

**Table 2:** Distribution of SNPs of Numb gene for study population

Varients		Total *N* (%)	Male *N* (%)	Female *N* (%)	*P* value
rs2108552 (SNP1)					
Genotype	C/C	281 (17.0)	174 (16.5)	107 (18.0)	0.732
	G/G	568 (34.4)	367 (34.8)	201 (33.7)	
	C/G	799 (48.6)	511 (48.7)	288 (48.3)	
Dominant model	CC	281 (17.0)	174 (16.5)	107 (18.0)	0.464
	CG + GG	1367 (83.0)	878 (83.5)	489 (82.0)	
Recessive model	GG	568 (34.4)	367 (34.8)	201 (33.7)	0.634
	CG + CC	1080 (65.6)	685 (65.2)	395 (66.3)	
Additive model	CG	799 (48.6)	511 (48.7)	288 (48.3)	0.922
	CC + GG	849 (51.4)	541 (51.3)	308 (51.7)	
Allele	C	1361 (41.3)	859 (40.8)	502 (42.1)	0.471
	G	1935 (58.7)	1245 (59.2)	690 (57.9)	
rs12435797 (SNP2)					
Genotype	T/T	532 (32.3)	323 (30.7)	209 (35.1)	0.111
	G/G	348 (21.1)	235 (22.3)	113 (19.0)	
	G/T	768 (46.6)	495 (47.0)	273 (45.9)	
Dominant model	TT	532 (32.3)	323 (30.7)	209 (35.1)	0.063
	GT + GG	1116 (67.7)	730 (69.3)	386 (64.9)	
Recessive model	GG	348 (21.1)	235 (22.3)	113 (19.0)	0.112
	GT + TT	1300 (78.9)	818 (77.7)	482 (81.0)	
Additive model	GT	768 (46.6)	495 (47.0)	273 (45.9)	0.660
	TT + GG	880 (53.4)	558 (53.0)	322 (54.1)	
Allele	T	1832 (55.6)	1141 (54.2)	691 (58.1)	0.031^*^
	G	1464 (44.4)	965 (45.8)	499 (41.9)	
rs1019075 (SNP3)					
Genotype	C/C	176 (10.7)	115 (10.9)	61 (10.2)	0.846
	T/T	848 (51.5)	536 (51.0)	312 (52.3)	
	C/T	624 (37.9)	401 (38.1)	223 (37.4)	
Dominant model	CC	176 (10.7)	115 (10.9)	61 (10.2)	0.660
	CT + TT	1472 (89.3)	937 (89.1)	535 (89.8)	
Recessive model	TT	848 (51.5)	536 (51.0)	312 (52.3)	0.585
	CT + CC	800 (48.5)	516 (49.0)	284 (47.7)	
Additive model	CT	624 (37.9)	401 (38.1)	223 (37.4)	0.778
	CC + TT	1024 (62.1)	651 (61.9)	373 (62.6)	
Allele	C	976 (29.6)	631 (30.0)	345 (28.9)	0.527
	T	2320 (70.4)	1473 (70.0)	847 (71.1)	
rs17781919 (SNP4)					
Genotype	C/C	1621 (98.4)	1034 (98.0)	587 (99.0)	0.133
	C/T	27 (1.6)	21 (2.0)	6 (1.0)	
Allele	C	3269 (99.2)	2089 (99.0)	1180 (99.5)	0.135
	T	27 (0.8)	21 (1.0)	6 (0.5)	

The *P* value of genotype was calculated by Fisher’s exact test

*P <0.05
3. Numb genotypes and lipid profile
  i. We found that the rs2108552 was significantly associated with plasma TC and LDL-C level in a dominant model, additive model, or recessive model before (All *P* < 0.05) and after multivariate adjustment of ethnicity, gender, age, smoking and obesity (All *P* < 0.05; Table 3). Further, the rs2108552 was significantly associated with plasma HDL-C level in a dominant model before (*P* = 0.000) and after multivariate adjustment (*P* = 0.012; Table 3).

**Table 3:** Associations between rs2108552 and lipid parameters

	Mean ± SD			Model 1			Model 2		
	Homozygous for wild allele (C)	Heterozygous	Homozygous for rare allele (G)	P (Dom)	P (Rec)	P (Add)	P (Dom)	P (Rec)	P (Add)
TC (mmol/L)	4.35 ± 1.24	4.34 ± 1.02	3.89 ± 1.01	0.000	0.042	0.006	0.000	0.007	0.015
LDL-C (mmol/L)	2.64 ± 0.96	2.70 ± 0.90	2.51 ± 0.83	0.007	0.009	0.030	0.000	0.004	0.011
HDL-C (mmol/L)	1.02 ± 0.56	1.02 ± 0.48	1.15 ± 0.67	0.000	0.213	0.120	0.012	0.913	0.083
TG (mmol/L)	1.99 ± 1.62	1.97 ± 1.41	1.91 ± 1.47	0.478	0.676	0.889	0.386	0.503	0.954

*TC* total cholesterol, *LDL-C* low density lipoprotein-cholesterol, *HDL-C* high density lipoprotein-cholesterol, *TG* triglycerides, *Dom* dominant model, *Rec* recessive model, *Add* additive model

Model 1: Unadjusted model; Model 2: Analysis of covariance adjusted for ethnicity, gender, age, smoking and obesity
  ii. By analyzing a dominant model, additive model, or recessive model, we found that the rs12435797 was significantly associated with plasma TC level before (All *P* < 0.05) and after multivariate adjustment of ethnicity, gender, age, smoking and obesity. (All *P* < 0.05; Table 4). In addition, we also found that rs12435797 was associated with HDL-C level in a dominant and an additive model (*P* = 0.001, *P* = 0.010). However, the difference did not remain statistically significant after multivariate adjustment.

**Table 4:** Associations between rs12435797 and lipid parameters

	Mean ± SD			Model 1			Model 2		
	Homozygous for wild allele (T)	Heterozygous	Homozygous for rare allele (G)	P (Dom)	P (Rec)	P (Add)	P (Dom)	P (Rec)	P (Add)
TC (mmol/L)	4.30 ± 1.28	4.36 ± 1.09	4.13 ± 1.02	0.000	0.005	0.004	0.007	0.006	0.016
LDL-C (mmol/L)	2.60 ± 0.96	2.70 ± 0.96	2.60 ± 0.80	0.171	0.279	0.030	0.808	0.545	0.527
HDL-C (mmol/L)	1.03 ± 0.66	1.01 ± 0.38	1.11 ± 0.67	0.001	0.494	0.010	0.874	0.791	0.833
TG (mmol/L)	1.94 ± 1.35	2.02 ± 1.60	1.93 ± 1.43	0.506	0.758	0.381	0.651	0.942	0.545

*TC* total cholesterol, *LDL-C* low density lipoprotein-cholesterol, *HDL-C* high density lipoprotein-cholesterol, *TG* triglycerides, *Dom* dominant model, *Rec* recessive model, *Add* additive model

Model 1: Unadjusted model; Model 2: Analysis of covariance adjusted for ethnicity, gender, age, smoking and obesity
  iii. We observed that the rs1019075 was significantly associated with plasma LDL-C and HDL-C level by analyses of a dominant model, additive model, or recessive model before (All *P* < 0.05) and after multivariate adjustment of ethnicity, gender, age, smoking and obesity (All *P* < 0.05; Table 5).

**Table 5:** Associations between rs1019075 and lipid parameters

	Mean ± SD			Model 1			Model 2		
	Homozygous for wild allele (C)	Heterozygous	Homozygous for rare allele (T)	P (Dom)	P (Rec)	P (Add)	P (Dom)	P (Rec)	P (Add)
TC (mmol/L)	4.25 ± 1.02	4.30 ± 1.23	4.22 ± 1.10	0.568	0.553	0.331	0.324	0.120	0.029
LDL-C (mmol/L)	2.68 ± 0.90	2.63 ± 0.91	2.57 ± 0.97	0.016	0.008	0.033	0.029	0.007	0.043
HDL-C (mmol/L)	1.02 ± 0.77	1.01 ± 0.46	1.08 ± 0.55	0.022	0.322	0.047	0.031	0.469	0.062
TG (mmol/L)	1.97 ± 1.45	1.95 ± 1.55	2.01 ± 1.47	0.662	0.936	0.719	0.367	0.707	0.353

*TC* total cholesterol, *LDL-C* low density lipoprotein-cholesterol, *HDL-C* high density lipoprotein-cholesterol, *TG* triglycerides, *Dom* dominant model, *Rec* recessive model, *Add* additive model

Model 1: Unadjusted model; Model 2: Analysis of covariance adjusted for ethnicity, gender, age, smoking and obesity
  iv. We found that rs17781919 was significantly associated with TC, LDL-C and HDL-C level (All *P* ≤ 0.000) and the difference remained statistically significant after (All *P* ≤ 0.001; (Table 6) multivariate adjustment of ethnicity, gender, age, smoking and obesity.

**Table 6:** Associations between rs17781919 and lipid parameters

	Mean ± SD		Model 1	Model 2
	Wild allele (T)	Rare allele (C)		
TG (mmol/L)	1.97 ± 1.50	1.66 ± 0.93	0.315	0.330
TC (mmol/L)	4.27 ± 1.11	3.46 ± 0.74	0.000	0.001
LDL-C (mmol/L)	2.64 ± 0.93	0.98 ± 0.55	0.000	0.000
HDL-C (mmol/L)	1.05 ± 0.49	1.92 ± 0.49	0.000	0.000

*TC* total cholesterol, *LDL-C* low density lipoprotein-cholesterol, *HDL-C* high density lipoprotein-cholesterol, *TG* triglycerides

Model 1: Unadjusted model; Model 2: Analysis of covariance adjusted for ethnicity, gender, age, smoking and obesity
  v. In addition, there exist no significant difference between different genotypes and the alleles of four SNPs in study cohorts for TG level (all *P* > 0.05, respectively; Tables 3–6).

## Discussion

### Findings

In this study, we have genotyped four kinds of SNP of Numb gene, and found that variation in Numb gene associates with cholesterol levels among Chinese subjects. This is the first endeavor to study the common allelic variant in Numb gene and its association with four types of lipid parameters.

Hypercholesterolemia has been observed to be the cause of multiple physiologic outcomes, such as coronary artery disease, diabetes, and obesity [34–36]. Previous study indicates that Numb gene may provide a therapeutic target for hypercholesterolemia [20]. Meanwhile, Numb genetic variant was directly responsible for the decreased LDL-C concentration [21]. However, few researches have been conducted regarding the relationship between polymorphism of Numb gene and lipid profile.

Our study shows that rs2108552 was significantly associated with plasma TC and LDL-C level by analyses of a dominant model, additive model, or recessive model. Such association was remained significant after multivariate adjustment. Further, individuals with the C allele of rs2108552 had significantly higher plasma TG and LDL-C level when compared with G allele. To our knowledge, a high level of LDL-C and TC concentration in plasma increase the risk of atherosclerotic cardiovascular disease. In previous study, we have found that rs2108552 of Numb gene was associated with CAD among Chinese subjects, and thus assumed CC genotype might be a risk genetic marker for CAD. Together, these results might provide convincing evidence for assuming people who carry C allele may have higher probabilities of suffering from atherosclerotic cardiovascular disease (CVD) than people who carry G allele of rs2108552 of Numb gene.

We also observed that C allele of SNP rs17781919 had significantly lower plasma TC level, LDL-C level and high HDL-C level when compared with T allele and further proved that this variation was associated with low level of LDL-C among large population. Moreover, these results also indicate that people who carry C allele reduces the risk of atherosclerotic cardiovascular disease (CVD) when compared to the people who carry T allele.

Similarly, we observed that the rs12435797 was significantly associated with plasma TC level, and rs1019075 was significantly associated with LDL-C and HDL-C level. Further, individuals with GG genotype of rs12435797 demonstrate low TC level compared with individuals with TT genotype, and individuals with TT genotype of rs1019075 also demonstrate low LDL-C level and high HDL-C level compared with individuals with CC genotype. Such association was also remained significant after multivariate adjustment. Therefore, we assumed that people who carry G allele of rs12435797 and T allele of rs1019075 may have lower probabilities of suffering from atherosclerotic cardiovascular disease (CVD) when compared to the people who carry T allele of rs12435797 and C of rs1019075 allele.

In addition, we have not observed a remarkable difference between TG level and three kinds of SNPs of Numb gene. This might indicate Numb gene does not have as strong correlation with TG concentration as it does with cholesterol concentration. Nevertheless, the relationship between TG concentration and Numb gene needs further study.

### Limitations and shortcomings

First, the source of subjects was limited to the First Affiliate Hospital of Xinjiang Medical University, and these subjects may possess some risk factors of cardiovascular disease and different kinds of lifestyles that both might positively influence lipid profile. Second, a longitudinal epidemiological study, over a reasonably long time span, is required to obtain evidence with higher quality and reliability.

## Conclusion

Our results indicate Numb gene rs2108552 and rs17781919 polymorphisms closely associate with cholesterol level, and this kind of association could be a crucial genetic marker and determinant in clinical research. Nevertheless, our study requires further epidemiological survey by resorting to large numbers of samples.

## Abbreviations

CAD: Coronary artery disease
LDL-C: Low density lipoprotein-cholesterol
HDL-C: High density lipoprotein-cholesterol
TC: Total cholesterol
TG: Triglycerides
SNPs: Single nucleotide polymorphisms
BMI: Body mass index
BUN: Blood urea nitrogen
Cr: Creatinine
PTB: Phosphotyrosine-binding domain
PRR: Proline-rich region domain
MAF: Minor allele frequency
DNA: Deoxyribonucleic acid
EDTA: Ethylenediaminetetraacetic acid
PCR: Polymerase chain reaction
SDS: Sequence detection systems
CVD: Cardiovascular disease
